# Portable, Battery-Operated, Low-Cost, Bright Field and Fluorescence Microscope

**DOI:** 10.1371/journal.pone.0011890

**Published:** 2010-08-04

**Authors:** Andrew R. Miller, Gregory L. Davis, Z. Maria Oden, Mohamad Reza Razavi, Abolfazl Fateh, Morteza Ghazanfari, Farid Abdolrahimi, Shahin Poorazar, Fatemeh Sakhaie, Randall J. Olsen, Ahmad Reza Bahrmand, Mark C. Pierce, Edward A. Graviss, Rebecca Richards-Kortum

**Affiliations:** 1 Beyond Traditional Borders, Rice University, Houston, Texas, United States of America; 2 Department of Bioengineering, Rice University, Houston, Texas, United States of America; 3 Department of Mycobacteriology, Pasteur Institute of Iran, Tehran, Iran; 4 Center for Molecular and Translational Human Infectious Diseases Research, The Methodist Hospital Research Institute, Houston, Texas, United States of America; Statens Serum Institute, Denmark

## Abstract

This study describes the design and evaluation of a portable bright-field and fluorescence microscope that can be manufactured for $240 USD. The microscope uses a battery-operated LED-based flashlight as the light source and achieves a resolution of 0.8 µm at 1000× magnification in fluorescence mode. We tested the diagnostic capability of this new instrument to identify infections caused by the human pathogen, *Mycobacterium tuberculosis*. Sixty-four direct, decontaminated, and serially diluted smears were prepared from sputa obtained from 19 patients suspected to have *M. tuberculosis* infection. Slides were stained with auramine orange and evaluated as being positive or negative for *M. tuberculosis* with both the new portable fluorescence microscope and a laboratory grade fluorescence microscope. Concordant results were obtained in 98.4% of cases. This highly portable, low cost, fluorescence microscope may be a useful diagnostic tool to expand the availability of *M. tuberculosis* testing at the point-of-care in low resource settings.

## Introduction

In developing countries, bright field microscopic evaluation of stained sputum smears remains the primary method for confirmation of *Mycobacterium tuberculosis* infection [1]. Although it is well known that the use of fluorescence microscopy can increase diagnostic sensitivity and reduce the time and expertise needed to interpret results, sophisticated microscope technologies are not widely available in developing countries, especially outside of centralized health centers [2], [3]. High capital costs, an unstable electrical supply, and harsh environmental conditions are major factors limiting the use of fluorescence microscopy outside of specialized facilities. As a result, patients must travel to such a facility for diagnosis, often resulting in delayed or deferred treatment, and thereby weakening the effectiveness of coordinated *M. tuberculosis* control programs [4], [5], [6].

Recent advances in fluorescence microscopy, including the use of low-cost light-emitting diodes (LEDs) as a light source, have helped to make the technology more widely available [1]. However, laboratory grade microscopes are costly and not optimized for portability [7]. As an example, the PrimoStar iLED (Zeiss, Oberkochen, Germany) costs $1875 USD in the 22 high-burden *M. tuberculosis* countries as identified by the World Health Organization and weighs 9.48 kg. A compact, light-weight, battery-operated, low-cost fluorescence microscope has the potential to enable diagnosis and screening of *M. tuberculosis* in remote settings with limited infrastructure, providing the proven effectiveness of fluorescence microscopy to the point-of-care.

To address this need, we developed the Global Focus microscope: a portable, battery-powered, inverted bright field and fluorescence microscope with up to 1000× magnification that can be manufactured for approximately $240 USD. In this report, we compare the diagnostic performance of the Global Focus microscope to a conventional fluorescence microscope (Nikon E400, Melville, NY) for the detection of *M. tuberculosis* bacilli from sputum smears obtained from clinical specimens.

## Materials and Methods

**Figure 1:**
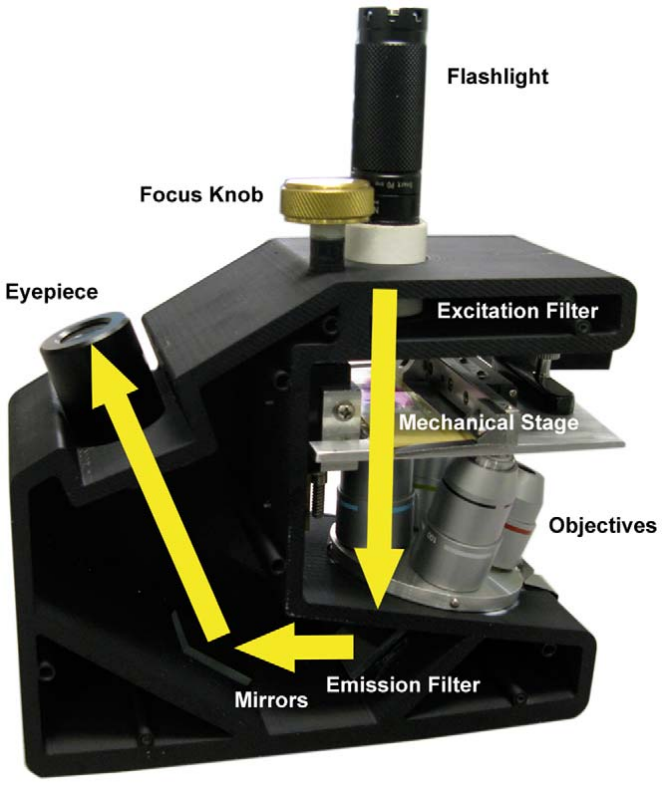
The Global Focus microscope. In this schematic, yellow arrows indicate the trans-illumination light path of the Global Focus microscope.

Figure 1 shows a photograph of the Global Focus microscope, illustrating the light path for imaging. A battery-powered LED flashlight serves as the light source. A flashlight containing a white LED is used for bright field imaging, while a flashlight containing a blue LED with a narrow spectral bandwidth excitation filter attached to the tip is used for fluorescence illumination. The flashlights are removable for ease of transportation. Spatial resolution was assessed measuring the average full width at half maximum of the point spread function of five sub-resolution fluorescent beads in the center of the field of view.

The diagnostic performance of the Global Focus microscope in fluorescence mode was compared to a Nikon E400 for the detection of *M. tuberculosis* bacilli from sputa. Sputum smears were derived from clinical specimens submitted for *M. tuberculosis* case finding under routine conditions at the Pasteur Institute (Tehran, Iran). Nineteen sputum samples were collected from 19 presumptive patients. Each sputum sample was used to generate a decontaminated preparation. Direct slides were prepared from 18 of the same specimens. For nine of the samples, an additional three preparations were generated: 1∶2, 1∶4, and 1∶8 dilutions from the decontaminated pellet. For the direct group, sputa were deposited onto the slides without additional treatment. For the decontaminated and diluted groups, sputa were chemically treated with sodium hypochlorate to lyse the cells and then centrifuged to concentrate the sample before application to the slide. Following the application of sputum, all slides were heat-fixed. Slides were stained using an auramine orange fluorescent dye kit (Medchem, Torrance, CA, USA) no more than one hour before evaluation. This study was reviewed by the Rice University IRB and found to be exempt from IRB review.

The 64 slides were randomized, assigned unique identification numbers, and reviewed by a blinded mycobacteriologist. During the evaluation, a randomly selected subset of 16 slides (25%) was concurrently reviewed by a blinded pathologist. All slides were evaluated first using the Global Focus microscope (Figure 1), and subsequently evaluated using a Nikon E400 microscope. Each slide was graded according to the International Union Against Tuberculosis and Lung Disease (IUATLD) scale, as follows: doubtful (1 to 2 AFB per 70 fields), 1+ (2–18 AFB per 50 fields), 2+ (4–36 AFB per 10 fields), 3+ (4–36 AFB per individual field), and 4+ (more than 36 AFB per individual field) [8]. Concordance of readings with the two microscopes was compared using the IUATLD scale. In addition, concordance of readings was compared when slides were judged as negative (IUATLD negative or doubtful) or positive (IUATLD 1+, 2+, 3+, 4+).

A Mann-Whitney U non-parametric analysis (SAS 9.2; Cary, NC, USA) was performed to test the hypothesis that the two microscopes yielded equivalent results using data from the direct and decontaminated groups.

## Results

**Figure 2:**
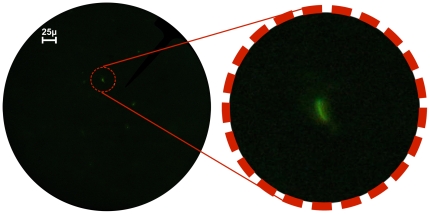
*M. tuberculosis* viewed with the Global Focus microscope. The image on the left is a photograph of *M. tuberculosis* bacilli stained with auramine orange, viewed with the Global Focus microscope at 400× magnification, and captured with a Canon G9 camera (F2.8, exposure: 1 second). The image on the right is a digital magnification detail of an *M. tuberculosis* bacillus.

The maximum spatial resolution of the Global Focus microscope in fluorescence mode was measured to be 0.8 µm at 1000× magnification [9]. Under these conditions, the resolution of the microscope is 3.2 times larger than the predicted resolution of 0.25 µm for the system. This discrepancy is likely due to the fact that the optical components were not corrected for field curvature and other aberrations. Moreover, no condenser lens was used. However, the parabolic reflector in the flashlight housing served to direct and shape light towards the sample. *M. tuberculosis* bacilli could be clearly resolved at 400× magnification, as seen in Figure 2.

Seventeen of the 19 decontaminated specimens examined with the two microscopes resulted in concordant evaluations (89.4% concordance). In two cases, samples judged as doubtful with the Nikon E400 microscope were judged as negative with the Global Focus microscope.

Fourteen of the 18 direct specimens examined with the two microscopes resulted in concordant evaluations (77.7% concordance). In one case, a sample judged as 1+ with the Global Focus microscope was judged as 3+ with the Nikon E400. In two cases, samples judged as doubtful with the Nikon E400 microscope were judged as negative with the Global Focus microscope. In one case, a sample evaluated as negative with the Nikon E400 microscope was classified as 1+ with the Global Focus microscope; the decontaminated preparation from this patient was evaluated as 1+ with both microscopes.

Twenty-six of the 27 diluted specimens examined with both microscopes resulted in concordant evaluations (96.3% concordance). In one case, a sample classified as doubtful with the Global Focus microscope was classified as negative with the Nikon E400 microscope.

Using IUATLD guidelines, concordant results were obtained in 56 of 64 specimens (87.5% concordance). When samples were evaluated as positive or negative, results with the two microscopes agreed in all but one case (98.4% concordance).

No statistical differences were measured between the Global Focus and Nikon E400 microscopes for detecting *M. tuberculosis* bacilli in the following groups: direct sputum smear (*n*_1_ = *n*_2_ = 18, H = 0.03, 1 d.f., p = 0.86); and the decontaminated smear (*n*_1_ = *n*_2_ = 19, H = 0.03, 1 d.f., p = 0.87).

## Discussion

We have developed a bright field and fluorescence microscope that provides an alternative to conventional fluorescence microscopy for the diagnosis of *M. tuberculosis* in sputum smears. The compact size (7.5×13×18 cm), minimal weight (1 kg), and minimal power requirements (two AA batteries) of the Global Focus microscope provide a favorable option for diagnostic settings with minimal laboratory infrastructure. This study indicates that evaluation of smears using the Global Focus microscope yields similar results as evaluation with a standard clinical laboratory grade fluorescence microscope. Importantly, the patient specimens tested herein were collected in a remote region of Iran, and therefore represent the prototypical samples that would be evaluated in the field with the Global Focus microscope. While future field studies are planned to evaluate the reliability and ease of use of this microscope, the results presented here serve as a proof of principle that diagnosis of *M. tuberculosis* is possible using low-cost, portable fluorescence microscopy.
